# Hemispheric asymmetries in hippocampal volume related to memory in left and right temporal variants of frontotemporal degeneration

**DOI:** 10.3389/fneur.2024.1374827

**Published:** 2024-04-29

**Authors:** Robert S. Hurley, Brittany Lapin, Stephen E. Jones, Anna Crawford, James B. Leverenz, Aaron Bonner-Jackson, Jagan A. Pillai

**Affiliations:** ^1^Department of Psychology, Cleveland State University, Cleveland, OH, United States; ^2^Department of Quantitative Health Sciences, Lerner Research Institute Cleveland Clinic, Cleveland, OH, United States; ^3^Center for Outcomes Research and Evaluation, Neurological Institute Cleveland Clinic, Cleveland, OH, United States; ^4^Department of Diagnostic Radiology, Imaging Institute Cleveland Clinic, Cleveland, OH, United States; ^5^Lou Ruvo Center for Brain Health, Neurological Institute Cleveland Clinic, Cleveland, OH, United States

**Keywords:** frontotemporal lobar degeneration, frontotemporal dementia, primary progressive aphasia, semantic dementia, Alzheimer’s disease, episodic memory, hippocampus, right temporal variant FTD

## Abstract

In addition to Alzheimer’s disease (AD), the hippocampus is now known to be affected in variants of frontotemporal degeneration (FTD). In semantic variant primary progressive aphasia (svPPA), characterized by language impairments, hippocampal atrophy is greater in the left hemisphere. Nonverbal impairments (e.g., visual object recognition) are prominent in the right temporal variant of FTD (rtvFTD), and hippocampal atrophy may be greater in the right hemisphere. In this study we examined the hypothesis that leftward hippocampal asymmetry (predicted in svPPA) would be associated with selective verbal memory impairments (with relative preservation of visual memory), while rightward asymmetry (predicted in rtvFTD) would be associated with the opposite pattern (greater visual memory impairment). In contrast, we predicted that controls and individuals in the amnestic mild cognitive impairment stage of AD (aMCI), both of whom were expected to show symmetrical hippocampal volumes, would show roughly equivalent scores in verbal and visual memory. Participants completed delayed recall tests with words and geometric shapes, and hippocampal volumes were assessed with MRI. The aMCI sample showed symmetrical hippocampal atrophy, and similar degree of verbal and visual memory impairment. The svPPA sample showed greater left hippocampal atrophy and verbal memory impairment, while rtvFTD showed greater right hippocampal atrophy and visual memory impairment. Greater asymmetry in hippocampal volumes was associated with larger differences between verbal and visual memory in the FTD samples. Unlike AD, asymmetry is a core feature of brain-memory relationships in temporal variants of FTD.

## Introduction

Bilateral hippocampal atrophy is a striking feature in the amnestic variant of Alzheimer’s disease (AD) (1), supporting the commonly-accepted role of the hippocampus in episodic memory (2, 3). The hippocampus is also affected in some forms of frontotemporal degeneration (FTD), the second most common class of younger-onset neurodegenerative diseases (4). The FTD syndrome known as semantic variant primary progressive aphasia (svPPA) usually results from TAR DNA-binding protein 43 (TDP-43) proteinopathy (5, 6). Atrophy in svPPA is concentrated in the anterior temporal lobes and is consistently more severe in the language-dominant left hemisphere, resulting in core impairments in language comprehension (7, 8).

Atrophy in svPPA also extends to medial aspects of the temporal lobe, including hippocampal volume loss that sometimes exceeds that seen in AD (9, 10). Anterior temporal atrophy in FTD eventually becomes bilateral, but in the case of svPPA remains asymmetrically leftward throughout most of the disease (9, 11, 12). Episodic memory in svPPA is often worse for verbal compared to nonverbal visual material (9, 13–15), and has been correlated with lower hippocampal gray matter intensities in both hemispheres (13), although neuropathology in left temporal language regions is also likely involved (5). The clinical relevance of right-hemispheric hippocampal preservation in svPPA is of significance but largely unexplored.

There is an increasing realization that a third or more of temporal FTD cases may instead be asymmetrically rightward (16, 17). As in svPPA, the right temporal variant of FTD (rtvFTD) is usually caused by TDP-43 proteinopathy (6). Unlike svPPA, where language symptoms predominate, a plethora of nonverbal impairments have been reported in rtvFTD, and there is an active debate in the field as to how these individuals should be diagnosed and characterized (17–19). Deficits in socioemotional functioning are prominent (6, 11), as are difficulties with visual object recognition (20). In particular, inability to recognize faces (prosopagnosia) is a frequently reported symptom in rtvFTD (19, 21–25). There are fewer studies of rtvFTD compared to svPPA, but in one report Chan and colleagues (17) found that anterior temporal atrophy in rtvFTD extended to the hippocampus, resulting in greater volume loss in the right hemisphere.

If in some respects (including hippocampal atrophy patterns) svPPA and rtvFTD are mirror images of one another (17, 21, 26), the relative severity of verbal and visual episodic memory deficits in these syndromes is important to clarify, given their potential clinical significance. Of the few studies comparing verbal and visual memory in rtvFTD and svPPA, results have been variable. Borghesani et al. (16) found that rtvFTD and svPPA had equivalent verbal impairments, but visual impairments were present only in rtvFTD. Younes et al. (6) found that both svPPA and rtvFTD had verbal and visual memory impairments, but did not differ in severity from one another in either domain. Pozueta et al. (27) also found no differences between svPPA and rtvFTD in either verbal or visual memory, but did not include a control group (making it difficult to evaluate the presence and severity of impairments). Yet another study found that visual memory was worse in svPPA than in rtvFTD (28). In summary, prior studies examining verbal and visual memory in rtvFTD have yielded variable results, and have not included examination of potential relationships between these domains of memory and hippocampal volumes in each hemisphere.

In the current study, we therefore examined verbal and visual memory, along with hippocampal volumes in each hemisphere (via MRI), in a retrospective consecutive series of individuals with svPPA and rtvFTD. A sample of individuals in the amnestic mild cognitive impairment (aMCI) stage of AD (29) was also assessed, allowing us to compare how AD versus FTD pathology is reflected in memory-hippocampal relationships. It is important to note that all assessments were performed within the first 2 years of symptoms. Finally, neurotypical control participants without neurodegenerative disease were also evaluated (prospectively), allowing us to quantify and more readily interpret the relative degree of memory loss and hippocampal volume losses among the individuals with FTD and aMCI.

Our primary hypothesis was that asymmetry in hippocampal volumes would be associated with differential performance across the two memory testing platforms (i.e., a verbal/visual split in scores). This hypothesis led to the following specific predictions for each group: (1) symmetrical bilateral hippocampal atrophy in aMCI will result in severe and roughly equivalent impairments in both memory domains, (2) asymmetrically leftward hippocampal atrophy in svPPA will result in greater verbal memory impairments, and (3) asymmetrically rightward atrophy in rtvFTD will result in greater visual memory impairments.

## Methods

### Participants

Individuals with FTD and aMCI were identified in a retrospective review of medical records at the Cleveland Clinic Lou Ruvo Center for Brain Health in Cleveland. These records included approximately 3,000 cases with cognitive complaints seen per year at our clinic, over each of the past 10 years.

The FTD samples (*n* = 7 svPPA and *n* = 8 rtvFTD) represented consecutive cases seen in our clinic who met study inclusion and diagnostic criteria. In order to compare the effects of FTD to AD pathology, an AD sample was also recruited. In an effort to roughly equate the clinical samples for disease severity, the inclusion criteria of 1–2 years of symptoms was imposed for AD as well, resulting in a sample in the amnestic mild cognitive impairment stage of AD in the aMCI stage of AD (*n* = 8). The aMCI sample was randomly selected to be matched to the FTD samples, representing the most recent cases who had similar demographic properties (age, sex, education).

Clinical diagnosis was established according to current criteria for the aMCI stage of AD (29), svPPA (7), and rtvFTD (19), as reviewed to consensus by a clinical team including a neuropsychologist (A.B.-J.), and two behavioral neurologists (J.A.P. and J.B.L.). Additional, inclusion criteria included having detailed neuropsychological testing, MRI scans and AD biomarkers to support diagnoses.

The svPPA and rtvFTD groups were differentiated based on visual inspection of the laterality of anterior temporal lobe atrophy. CSF biomarkers included abeta-42, total-tau, phosphorylated-tau181 (p-tau181), and abeta42/p-tau181 ratios using the Athena Diagnostics ADmark® ELISA test, as indicators of AD pathology. Imaging biomarkers included FDG-PET scans and Amyvid™ PET amyloid scans. Although rtvFTD is sometimes conceptualized as including the behavioral variant of FTD when associated with predominant anterior temporal atrophy (6), any individuals meeting criteria for the behavioral phenotype (30) were excluded from the current study.

A control group (*n* = 22) was recruited prospectively for this study, to aid interpretation of results by anchoring relative losses of memory and hippocampal volumes in the FTD and aMCI groups to control values. The control participants were recruited and completed behavioral testing at Cleveland State University. They were screened to be matched to the FTD and aMCI samples in terms of demographic variables (age, sex, handedness, education, race/ethnicity). A history of medical conditions that could affect cognition was exclusionary, including cognitive impairment from neurodegenerative disease, developmental impairments, strokes, epilepsy, or any other self-reported neurological, psychiatric, or pharmacological conditions that may affect language, cognition, or study performance. They were required to have corrected vision sufficient to read and hearing sufficient to engage in conversation. All controls performed within normative (asymptomatic) ranges on the brief version of the Mini Mental State Examination-2 (31), and had delayed recall scores within normative ranges on the Hopkins Verbal Learning Test-Revised (32) and Brief Visuospatial Memory Test-Revised (33). No AD biomarkers were available for the control group. The control group completed MRI scans on the same scanner using the same imaging protocol used for the clinical samples (see Imaging section below).

Verbal memory was tested via the Logical Memory subtest from the Wechsler Memory Scale (34), Hopkins Verbal Learning Test-Revised, or Rey Auditory Verbal Learning Test (35). Visual memory was tested by the Visual Reproduction subtest from Wechsler Memory Scale (third and fourth editions (34, 36)) or Brief Visuospatial Memory Test-Revised. Raw delayed recall scores were converted to standard scores (mean = 100, standard deviation = 15) based on sex and age-specific normative data from each measure, in accordance with the scoring instructions for each test. The Boston Naming Test (37) was administered as a measure of language and object recognition, with scores also standardized (mean = 100, standard deviation = 15) using sex, age, and education-specific norms. Total scores from the Montreal Cognitive Assessment (MoCA) (38) were examined as a global measure of cognitive functioning in the patient groups (not administered to controls).

All participants were required to be primary English speakers.

### Imaging

All participants completed MRI scanning at the Cleveland Clinic, on a 3T Siemens Skyra (Erlangen, Germany) using a 20-channel head coil. Volumetric data were derived using a T1 MPRAGE sequence from the Alzheimer’s Disease Neuroimaging Initiative protocol. The images were post-processed using NeuroQuant™ software (Cortech Labs Inc., La Jolla, California), including quantification of hippocampal volumes in each hemisphere, which are reported as a percentage of each participant’s total intracranial volume (ICV). Whole brain volumes (also expressed as a percentage of ICV) are reported as a measure of overall atrophy.

### Data strategy

Individuals with svPPA and rtvFTD represent a minority of individuals treated at our clinic, and small sample sizes were obtained. As such, our study design placed emphasis on the directionality of means and deviation of each clinical sample from control values, as we did not have sufficient power to compare groups via interaction terms in full factorial models. Nonparametric tests were employed to be more robust to small sample sizes. Cohen’s d effect sizes were calculated where values >0.2, >0.5, and > 0.8 indicate small, medium, and large effects, respectively (39).

Demographic and clinical characteristics were compared across all groups. Continuous variables were compared using Kruskal-Wallis test with posthoc Wilcoxon rank sum tests, while categorical variables were compared using chi-square test.

To clearly delineate how the three clinical samples deviated from typical performance in each memory modality, “memory loss” values were constructed by expressing each standardized test score in terms of standard deviations from control values (i.e., a z-score conversion using the mean and standard deviation from the control sample). Similarly, “hippocampal volume loss” values were constructed for each clinical group, expressing each participant’s ICV-corrected volumes in standard deviations from the control sample. Memory loss and hippocampal volume loss were compared between clinical samples using effect sizes and Wilcoxon rank sum tests.

Our overall hypothesis was that asymmetry in hippocampal atrophy would be associated with differential performance across verbal and visual memory modalities. To evaluate this association, asymmetry in hippocampal volumes was quantified by calculating a “hippocampal difference score,” subtracting left from right volumes. Likewise, a “memory difference score” was calculated by subtracting verbal from visual test values. Spearman correlation coefficients were calculated between these two difference scores across the three clinical samples, and in the two FTD groups where we anticipated asymmetry. Given the *a priori* and directional nature of our hypothesis, a one-tailed test criteria was employed.

Statistical significance was established throughout at *p* < 0.05. Due to small sample sizes, emphasis was placed on magnitudes of effect and there was no formal adjustment for multiple comparisons. All statistical analyses were conducted using SAS version 9.4 (SAS Institute Inc., Cary NC).

## Results

### Group characteristics

The demographic and clinical composition of each group is shown in Table 1. The groups did not significantly differ in terms of age or education (*p* > 0.05 on Kruskal-Wallis tests); and also did not significantly differ in sex, handedness, or race/ethnicity (*p* > 0.05 on all chi-square tests). Given the small sample sizes included in this study, this does not necessarily mean the groups were fully-matched on these variables, but rather that no flagrant mismatches were detected via inferential comparisons.

**Table 1:** Demographic and clinical characteristics by patient sample.

	Control	aMCI	svPPA	rtvFTD
Sample size, *n*	22	8	7	8
Age, mean (SD)	68.5 (5.9)	69.0 (6.5)	71.4 (8.5)	63.4 (10.1)
Female, *n* (%)	12 (54.6)	4 (50.0)	2 (28.6)	2 (25.0)
Right-handed, *n* (%)	22 (100.0)	8 (100.0)	7 (100.0)	7 (87.5)
White, *n* (%)	20 (90.9)	7 (87.5)	6 (85.7)	8 (100.0)
Black, *n* (%)	2 (9.1)	1 (12.5)	1 (14.3)	0
Hispanic, *n* (%)	1 (4.6)	0	0	0
Years of education, mean (SD)	17.1 (1.6)	15.9 (2.9)	15.4 (4.1)	16.4 (2.6)
Years of symptoms, median (q1, q3)	–	2.0 (2.0, 2.0)	2.0 (1.0, 2.0)	2.0 (1.0, 2.0)
CSF abeta42, mean (SD)	–	272.9 (84.3)*	1011.7 (260.0)	684.3 (244.1)
CSF total-tau, mean (SD)	–	494.8 (154.9)	468.2 (82.3)	345.0 (45.9)*
CSF p-tau181, mean (SD)	–	75.5 (14.3)	79.8 (10.5)	45.2 (14.1)*
CSF abeta42/p-tau181 ratio, mean (SD)	–	3.8 (1.7)*	13.1 (5.0)	16.0 (5.5)
MoCA total scores, mean (SD)	–	18.6 (6.3)	15.6 (5.8)	20.3 (4.9)
Boston Naming Test, mean (SD)	106.1 (11.0)*	93.3 (19.5)*	61.7 (9.8)	67.9 (9.8)
Verbal memory, mean (SD)	93.8 (12.9)*	53.5 (9.9)	56.6 (10.6)	64.4 (14.9)
Visual memory, mean (SD)	104.3 (13.6)*	62.4 (13.4)	75.9 (16.9)	66.4 (20.1)
Whole brain volume (%ICV), mean (SD)	76.5 (2.3)	73.1 (6.4)	72.1 (3.5)	70.0 (3.5)
Left hippocampal volume (%ICV), mean (SD)	0.25 (0.03)*	0.23 (0.02)	0.19 (0.03)	0.21 (0.03)
Right hippocampal volume (%ICV), mean (SD)	0.27 (0.03)*	0.23 (0.03)	0.25 (0.03)	0.17 (0.03)

*: *p* < 0.05 vs. all other samples. CSF concentration values are reported in pg./ml. Verbal memory, visual memory, and Boston Naming Test values are reported in standardized scores (mean = 100, standard deviation = 15). Hippocampal volumes are expressed as a percentage of ICV. aMCI, amnestic variant mild cognitive impairment stage of Alzheimer’s disease; svPPA, semantic variant primary progressive aphasia; rtvFTD, right temporal variant frontotemporal degeneration; SD, standard deviation; q1, first quartile; q3, third quartile; CSF, cerebrospinal fluid (markers); p-tau181, phosphorylated tau181; ICV, intracranial volume.

CSF data for AD biomarkers were available for 7/8 members of the aMCI group, 3/7 in the svPPA group, and 7/8 in the rtvFTD group (additional biomarkers including brain FDG-PET and Amyvid™ PET supported diagnosis in others). The Kruskal-Wallis tests were significant for all CSF biomarkers (all *p* < 0.01), indicating group differences. Pairwise comparisons showed that abeta-42 concentrations and abeta-42/p-tau181 ratios were significantly lower in the aMCI group compared to the two FTD groups (*p* < 0.0.05; *d* range-2.25 to-4.95). The rtvFTD group showed significantly lower total-tau and p-tau181 concentrations compared to the other two clinical samples (all *p* < 0.05; *d* range-1.31 to-2.6).

Amyvid™ PET amyloid scans were available for one participant with aMCI (who was amyloid positive), one participant with svPPA (amyloid negative), and one participant with rtvFTD (amyloid negative). FDG-PET scans were available for three participants with svPPA and one participant with rtvFTD, all showing characteristic anterior temporal hypometabolism, with preserved regional metabolism in posterior cingulate and parietotemporal regions that are affected in AD. These biomarker results suggest that AD and non-AD neuropathologies were likely dominant in the aMCI and FTD groups, respectively.

MoCA total scores did not differ across the three clinical groups (*p* = 0.21), but Boston Naming Test scores did significantly differ across groups (*p* < 0.001). Pairwise comparisons showed that naming was most impaired in the svPPA and rtvFTD groups (who did not differ from one another), with lesser naming impairment in the aMCI group (all *p* < 0.05). This fits with previous findings that confrontation naming is greatly disrupted by the presence of single-word comprehension deficits and/or visual agnosia in svPPA and rtvFTD, as successful naming requires recognition of the object and linkage with its corresponding lexical representation (19, 40, 41).

All three clinical groups showed significantly lower memory scores compared to controls, on both the verbal and visual memory modalities (all *p* < 0.05; *d* range 1.98 to 3.3). All three clinical groups also showed significantly lower hippocampal volumes compared to controls (all *p* < 0.05, *d* range 1.09 to 3.18), except the svPPA group, which did not reach conventional statistical significance for right hippocampal values when compared to controls (*p* = 0.10, *d* = 0.78). Differences between clinical groups are further examined in the following sections.

Whole brain volumes in the rtvFTD group were significantly lower than controls (*p* < 0.001, d = 2.46), and aMCI trended towards being lower than controls (*p* = 0.057; *d* = 0.99). The svPPA whole brain volumes, however, did not differ from controls (*p* = 0.35, *d* = 1.32). There were no significant differences in whole brain volumes between clinical groups (*p* = 0.57, *d* range 0.15 to 0.61).

### Memory comparisons across clinical samples

**Figure 1:**
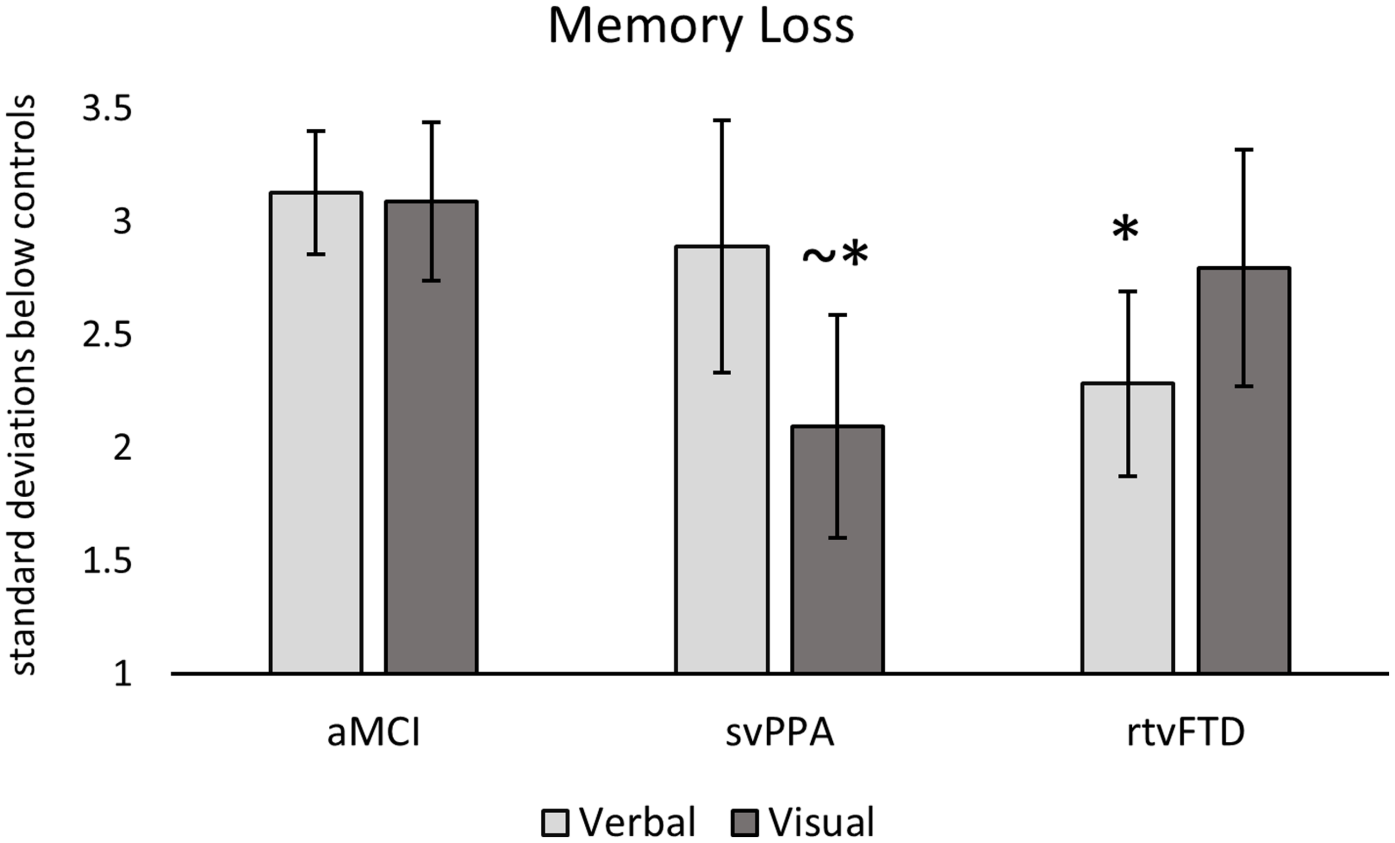
Memory loss in clinical samples. Standardized verbal and visual memory scores of each clinical sample are re-expressed in terms of standard deviations below the control sample values (i.e., z-scores, with standard error bars). Larger values therefore represent lower memory scores. Significant differences between clinical samples are denoted. Visual memory loss was less severe in svPPA compared to aMCI, while verbal memory loss was less severe in rtvFTD compared to aMCI. ~*: *p* ≤ 0.10 vs. aMCI; *: *p* ≤ 0.05 vs. aMCI. aMCI, amnestic variant mild cognitive impairment stage of Alzheimer’s disease; svPPA, semantic variant primary progressive aphasia; rtvFTD, right temporal variant frontotemporal degeneration.

The memory loss scores for each clinical sample are shown in Figure 1, expressed as standard deviations from control values. The aMCI group showed severe and symmetrical memory loss in both verbal and visual domains. The svPPA group showed a similar level of verbal memory loss to the aMCI group (*p* = 0.36; *d* = 0.30), but less visual memory loss (*p* = 0.10; *d* = 0.89). The rtvFTD group demonstrated the opposite pattern, with a similar degree of visual memory loss to the aMCI group (*p* = 0.83; *d* = 0.23) but less verbal memory loss than aMCI (*p* = 0.037; *d* = 0.86). Thus, the svPPA and rtvFTD groups showed similar memory loss to aMCI in their relative areas of weakness (verbal and visual material, respectively), and in their relative areas of preservation they showed loss when compared to controls (Table 1) but less severe than seen in aMCI.

### Hippocampal volume comparisons across clinical samples

**Figure 2:**
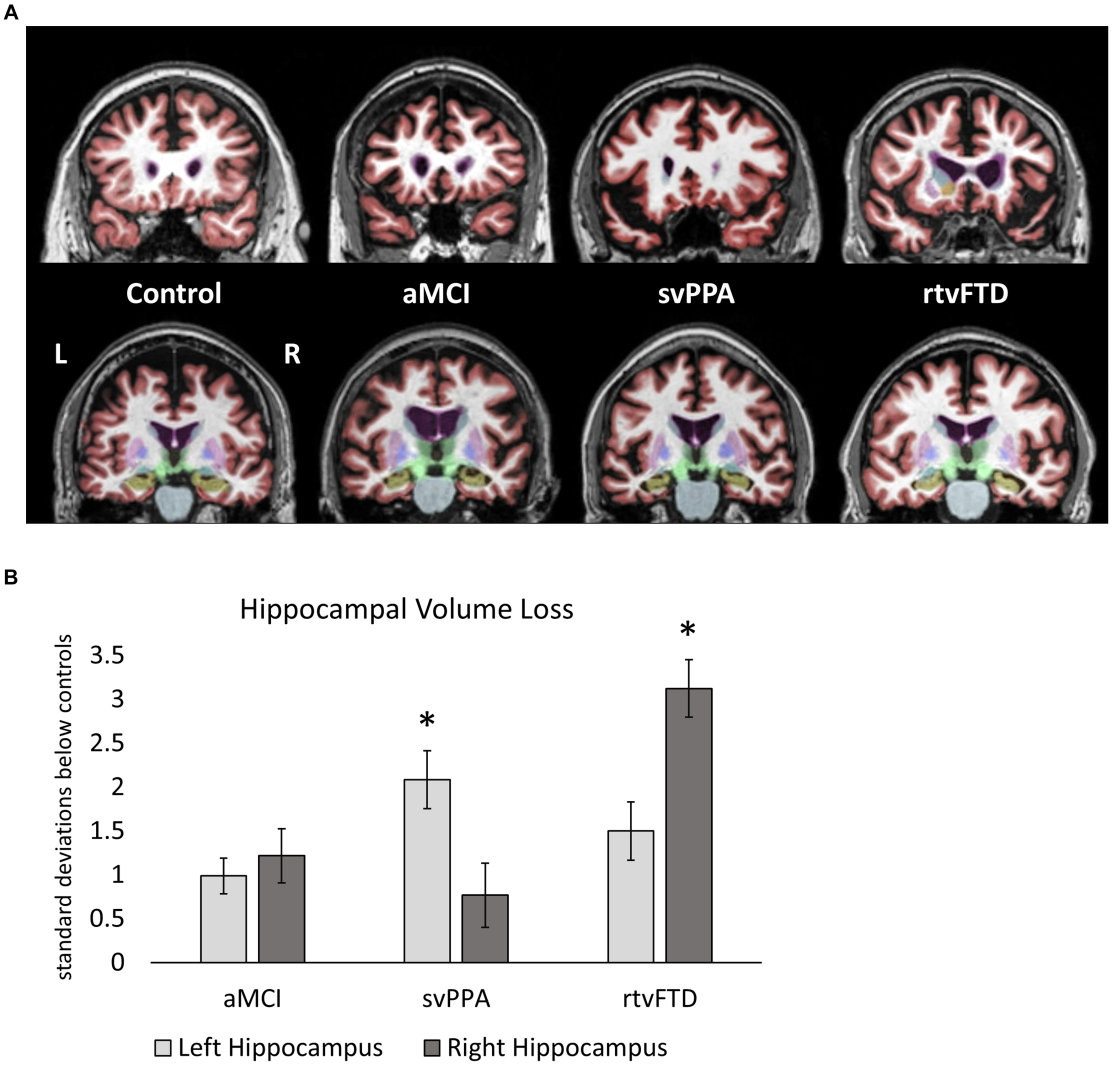
**(A)** Representative MRI scans from each group showing the anterior temporal lobes (top row) and the hippocampi (bottom row). The left hemisphere is on the left side in these images (non-radiologic convention). Participants with median hippocampal volumes in each group were identified, and a coronal slices through anterior temporal cortex (in red) and the hippocampus (in beige) are shown for each. Temporal atrophy is bilateral and roughly symmetrical in aMCI, and is asymmetrically leftward in svPPA and rightward in rtvFTD. **(B)** Hippocampal volume loss in clinical samples. Hippocampal volumes (as a percentage of ICV) are expressed in terms of standard deviations below the control sample values. Larger values therefore represent lower hippocampal volumes. Significant differences between clinical samples are denoted. Left hippocampal volume loss was greater in svPPA compared to aMCI, while right hippocampal volume loss was greater in rtvFTD compared to aMCI. *: *p* ≤ 0.05 vs. aMCI. aMCI, amnestic variant mild cognitive impairment stage of AD; svPPA, semantic variant primary progressive aphasia; rtvFTD, right temporal variant frontotemporal degeneration; ICV, intracranial volume.

Representative MRI scans from members of each group are shown in Figure 2A. Hippocampal volume loss scores for each clinical sample are shown in Figure 2B, expressed as standard deviations from control values. The svPPA group showed a greater volume loss than the aMCI group in the left hippocampus (*p* = 0.019; *d* = 1.5), while volumes in the right hemisphere did not differ from aMCI (*p* = 0.41; *d* = 0.49). The rtvFTD group showed the opposite pattern, with greater volume loss in the right (*p* < 0.01; *d* = 2.12) but not left hemisphere (*p* = 0.22; *d* = 0.65), compared to aMCI.

### Association between hippocampal asymmetry and memory

**Figure 3:**
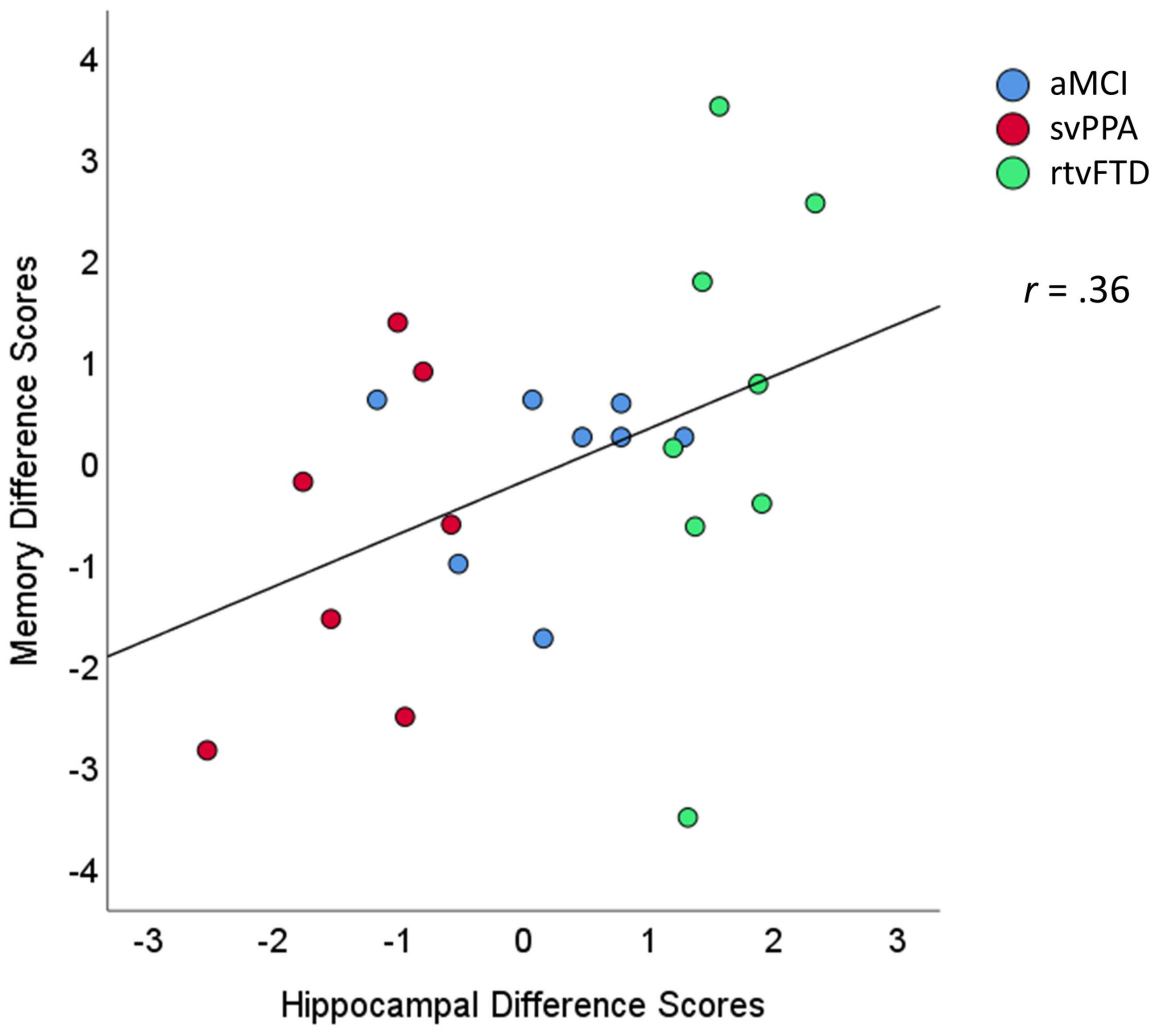
Scatterplot of the association between hippocampal and memory difference scores. The hippocampal difference scores were calculated as volume loss in the left minus the right hemisphere (e.g., negative values reflect greater loss in the left hemisphere). The memory difference scores reflect scores on verbal minus visual memory tests. Units are expressed in standard deviations from control values. aMCI, amnestic variant mild cognitive impairment stage of AD; svPPA, semantic variant primary progressive aphasia; rtvFTD, right temporal variant frontotemporal degeneration.

A nonparametric Spearman correlation between the two difference scores (left–right hippocampal volumes and verbal-visual memory scores) was significant when examined across all three clinical samples (Figure 3; *r* = 0.36; *p* = 0.048). This correlation was also significant when examined solely among the two FTD groups with significant asymmetry: the svPPA and rtvFTD groups (*r* = 0.48; *p* = 0.037).

## Discussion

### Summary

The svPPA, rtvFTD, and aMCI groups all showed lower memory scores for both verbal and visual material, compared to neurotypical controls. All clinical samples also showed lower hippocampal volumes, in both hemispheres, compared to controls. When memory scores and hippocampal volumes in the clinical samples were re-expressed as “loss score” differences from control values, distinct brain-memory relationships in FTD became apparent which differed from those seen in aMCI, based on areas of relative preservation and loss.

In aMCI, symmetrical hippocampal atrophy was associated with equally poor memory for verbal and visual material. In FTD, our primary hypothesis was supported, such that asymmetry in hippocampal volumes was correlated with a disparity in scores between the verbal and visual memory testing platforms. Hippocampal volume loss in svPPA exceeded that of aMCI in the more atrophic left hemisphere, and memory loss in svPPA was greater for verbal than for visual material. The opposite pattern was observed in rtvFTD, such that right hippocampal volume loss was maximal (also exceeding that seen in aMCI), and memory loss in rtvFTD was greater for visual material. Both FTD groups showed memory loss equivalent to aMCI in their more affected memory domain (verbal for svPPA, visual for rtvFTD), while performance in their relatively preserved domain (visual for svPPA, verbal for rtvFTD) was intermediate to aMCI and controls. These findings reinforce the notion that in some respects svPPA and rtvFTD are mirror images of one another (21, 26).

These findings suggest that the relationship between hippocampal pathology and episodic memory in FTD differs from that seen in AD; lateralized atrophy in FTD is associated with domain-specific memory loss, leaving relative areas of preservation in memory.

### Limitations

The retrospective design employed in this study included small sample sizes. Emphasis was placed on the directionality of means and deviation of the FTD and aMCI groups from control values. As such, the results from this study can be considered as proof of concept rather than definitive evidence, demonstrating how domain-specific memory specializations in each hemisphere may play out with regards to temporal FTD variants. The current findings would benefit from replication with larger samples, employing more robust statistical models with group interaction terms.

With larger samples, it may also be possible to probe for additional factors that may mediate the apparent relationship between hippocampal asymmetry and memory domain observed in this study. For example, medial temporal lobe atrophy in svPPA does not occur in isolation, but alongside temporopolar and lateral temporal atrophy in the left-hemispheric language network. It is therefore possible that poor recall of verbal material is primarily driven by dysfunction in the language network rather than the episodic memory network (13). Likewise, medial temporal atrophy in rtvFTD co-occurs with atrophy in the object recognition network, including the right temporal pole and fusiform (19, 21, 42). Larger studies with targeted memory experiments could help to disentangle which networks are most directly responsible for apparent memory deficits in FTD.

Inclusion in this study was restricted to individuals with 1–2 years of symptoms. This yields certain strength to our study design: emergent anatomic changes are constrained within this early time frame (facilitating targeted group comparisons), and symptoms may still be relatively isolated to key domains of cognition. Restricting inclusion to initial years of symptoms does not, however, necessarily equate the groups in terms of their current severity of neuropathology burden, or the severity of those resultant symptoms. Although our retrospective study records did not include an overall measure of dementia severity (e.g., the Clinical Dementia Rating Scale (43)), the patient groups did not significantly differ in global cognition (as measured by MoCA total scores), which is highly correlated with functional impairment (44).

### Future directions

Unlike aMCI and AD, asymmetry is a core feature of brain-memory relationships in temporal variants of FTD. Lateralized hippocampal atrophy in our study was correlated with domain-specific memory impairments in FTD, supporting the inclusion of both verbal and visual memory tests during clinical assessment. Right hippocampal volume loss in svPPA was on par with that seen in aMCI, but visual memory impairments were not as severe. Conversely, left hippocampal volume loss in rtvFTD was similar to aMCI, but verbal memory was less affected. Further work is needed to determine why hippocampal atrophy triggered by AD pathology is generally more detrimental to episodic memory than atrophy caused by FTD pathology. It may be, for example, that posterior aspects of the hippocampus and/or other components of the wider episodic memory network remain relatively intact in FTD, as compared to AD (45, 46). It is also possible that as both verbal and visual domains of memory are affected in AD (unlike FTD where at least one domain is relatively spared), memory loss in AD is strikingly apparent in clinical evaluations. Future studies employing network analyses (rather than focusing on a single structure, as we did in this study) may reveal the extent to which verbal and visual memory are the product of distributed networks, and uncover additional mechanisms by which those functions are disrupted in diseases such as FTD.

In this study we focused on delayed recall, which represents only one aspect of episodic memory. Changes in autobiographical memory have been reported in FTD (47), but few studies have examined memory in detail specifically in the rtvFTD variant. Future studies may help to reveal to what extent other aspects of episodic memory may be affected in rtvFTD, such as immediate recall, recognition, and remote memory.

Even though TDP-43 is considered a common etiology of svPPA and rtvFTD, underlying tau pathology has also been reported in these variants (48). However, lower levels of p-tau in our rtvFTD cohort suggest that it is likely that our specific rtvFTD cases are more likely to be enriched for TDP-43 pathology than the svPPA cases (49). In cases where FTLD-tau is the underlying etiology for svPPA or rtvFTD, one can hypothesize that the episodic memory relationships noted with respect to TDP-43 related hippocampal atrophy (50) could be less prominent. This hypothesis needs to be carefully evaluated in future clinico-pathological studies.

It would also be helpful to achieve some sort of consensus as to the nature of the rtvFTD syndrome, and ultimately to settle on standardized diagnostic criteria. While there is general agreement that rtvFTD manifests as a bouquet of nonverbal symptoms, it remains unclear as to whether there is a core or signature impairment. The current findings suggest that nonverbal impairments in visual object processing are an important part of the syndrome. This fits with the finding that prosopagnosia is one of the most common symptoms in rtvFTD (19, 21). Impairments in object recognition (agnosia) are also apparent in svPPA, in cases where anterior temporal atrophy has become appreciable in the right hemisphere (i.e., bilateral) (42).

The rtvFTD syndrome has also been reported, however, to profoundly affect socioemotional function, person-specific knowledge, interoception, and reward processing (6, 18, 26). In keeping with these symptoms, some groups have adopted a wider view of rtvFTD, such that it also encompasses the behavioral variant of FTD when caused by predominant right anterior temporal atrophy (6). Others have questioned whether rtvFTD would be better conceptualized as manifesting in multiple distinct phenotypes rather than a single clinicopathologic entity (18, 19). Future studies may help to deepen our understanding of the relationships between proteinopathy, polymorphisms/mutations, structural changes, functional changes, and cognitive symptoms in rtvFTD, as has been the case in svPPA.

## Data availability statement

The raw data supporting the conclusions of this article will be made available by the authors, without undue reservation.

## Ethics statement

The studies involving humans were approved by Institutional Review Boards of the Cleveland Clinic and Cleveland State University. The studies were conducted in accordance with the local legislation and institutional requirements. Written informed consent for participation in this study was provided by the participants’ legal guardians/next of kin. Written informed consent was obtained from the individual(s) for the publication of any potentially identifiable images or data included in this article.

## Author contributions

RH: Resources, Investigation, Writing – original draft, Project administration, Funding acquisition, Data curation, Conceptualization. BL: Visualization, Formal analysis, Writing – review & editing, Data curation. SJ: Writing – review & editing, Software, Project administration, Investigation. AC: Writing – review & editing, Investigation, Formal analysis, Data curation. JL: Writing – review & editing, Resources. AB-J: Writing – review & editing, Investigation, Data curation. JP: Writing – review & editing, Supervision, Resources, Project administration, Methodology, Investigation, Funding acquisition, Data curation, Conceptualization.
